# Phenotyping of immune and endometrial epithelial cells in endometrial carcinomas revealed by single-cell RNA sequencing

**DOI:** 10.18632/aging.202288

**Published:** 2021-01-10

**Authors:** Yu-e Guo, Yiran Li, Bailian Cai, Qizhi He, Guofang Chen, Mengfei Wang, Kai Wang, Xiaoping Wan, Qin Yan

**Affiliations:** 1 Clinical and Translational Research Center, Shanghai First Maternity and Infant Hospital, Tongji University School of Medicine, Shanghai, China; 2 Department of Gynecology, Shanghai First Maternity and Infant Hospital, Tongji University School of Medicine, Shanghai, China; 3 Department of Pathology, Shanghai First Maternity and Infant Hospital, Tongji University School of Medicine, Shanghai, China

**Keywords:** single-cell RNA sequencing, endometrial carcinoma, immune microenvironment, macrophage activation model, endometrial epithelial cells

## Abstract

Tumors are complex ecosystems harboring multiple cell types which might play a critical role in tumor progression and treatment response. The endometrial epithelial cell identities and immune microenvironment of endometrial carcinoma (ECC) are poorly characterized. In this study, a cellular map of endometrial carcinoma was generated by profiling 30,780 cells isolated from tumor and paratumor tissues from five patients using single-cell RNA sequencing. 7 cell types in lymphocytes, 7 types in myeloid cells and 3 types in endometrial epithelial cells were identified. Distinct CD8^+^ T cell states and different monocyte-macrophage populations were discovered, among which exhausted CD8^+^ T cells and macrophages were preferentially enriched in tumor. Both CD8^+^ T cells and macrophages comport with continuous activation model. Gene expression patterns examination and gene ontology enrichment analysis of endometrial epithelial cells revealed 3 subtypes: stem-like cells, secretory glandular cells and ciliated cells. Overall, our study presents a view of endometrial carcinoma at single-cell resolution that reveals the characteristics of endometrial epithelial cells in the endometrium, and provides a cellular landscape of the tumor immune microenvironment.

## INTRODUCTION

Tumors are complex ecosystems characterized by extensive heterogeneity which plays a critical role in tumor progression and treatment response [1]. The tumor microenvironment (TME) consists of malignant cells and genetically stable stromal cells. Cancer cells are heterogeneous because of genetic diversification. Similarly, stromal cells also form heterogeneous cellular compositions by combining many different cell types with a range of biological roles [2]. Tumor cellular diversity is both a challenge and an opportunity for cancer diagnosis and treatment. Increasing cancer therapies targeting the TME such as immunotherapies have been developed to complement the traditional treatment options. However, TME diversity influences treatment response of targeted agents resulting in inconsistent outcomes among patients [3]. It is therefore essential to distinguish the specific cellular components in terms of morphological and phenotypic profiles as well as characterize the interactions between the diverse cell types.

Endometrial cancer (EC) is a common gynecologic tumor whose incidence is increasing [4]. Endometrioid carcinoma (ECC) is the most common type of endometrial carcinoma, accounting for approximately 85% of cases [5]. Most ECC patients can be diagnosed at an early stage and be treated successfully after hysterectomy. However, some young patients may have a need of fertility preservation, so conventional surgical treatment is not the best option [6, 7]. In addition, some patients are too old to tolerate surgery or its side effects. As such, more effort needs to be put into promoting trials that will improve more treatment options for patients. Moreover, novel biomarkers for prediction of treatment responses and clinical decisions are also needed. TME is a fertile ground for the development of novel therapies and is thus a target of expanding EC studies [8]. For example, pembrolizumab demonstrated a favorable safety profile and durable antitumor activity in a subgroup of patients with PD-L1 positive endometrial cancer as an anti PD-1monoclonal antibody [9]. Apart from tumor PD-L1 expression correlating with pembrolizumab response, other factors from subsets of malignant cells and the microenvironment also play essential roles [10]. Illuminating the spectrum of immune and other cell states of EC can therefore be helpful in understanding how the TME influence tumor behavior.

Single-cell RNA-sequencing (scRNA-seq) technology enables cell population profiling of tumors at single-cell resolution [11]. For instance, one recent study on lung tumor assembled a comprehensive catalog of the complex TME by characterizing the phenotype and co-optive behavior of stromal cell. These findings shed new light into lung cancer biology [12]. Advances in single-cell sequencing technologies such as those of aqueous droplets have enabled researchers to simultaneously sequence thousands of cells in a biopsy sample to obtain large datasets [13]. Further to this, multiple bioinformatics and algorithmic approaches have been developed to analyze these datasets as well as identify cell types by clustering scRNA-seq data while reducing their technological noise [14]. These advancements have allowed for the assessment of intra- and inter-tumoral heterogeneities of both stromal and cancer cell types. Moreover, they also help identify the states of these cells in the complex EC tumor cellular ecosystem.

In this study, bioinformatics analysis of the single-cell transcriptome was done. This data was used to reveal complexities of the endometrioid carcinoma’s (ECC) immune and the endometrial epithelial cellular composition as well as their differences with their counterparts residing in paratumor tissues.

## RESULTS

### Single-cell RNA-seq and cell typing in endometrial carcinomas and paratumor samples

**Figure 1:**
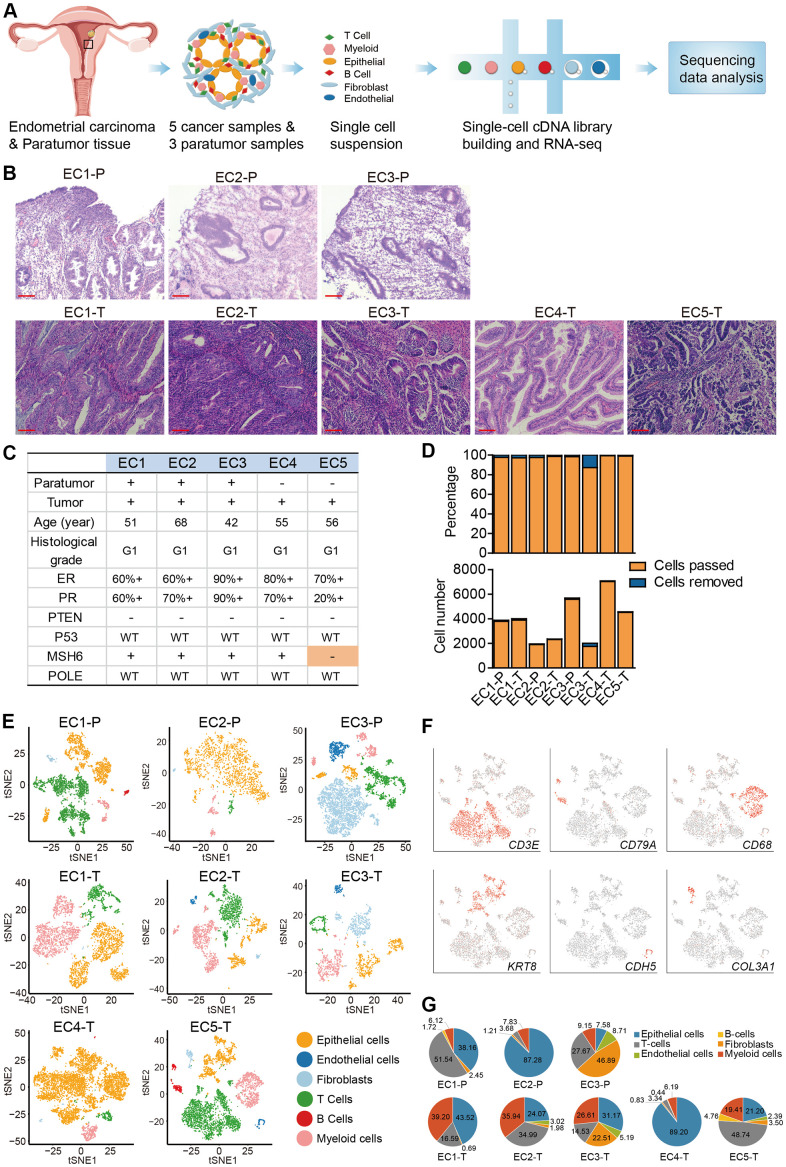
**Diversity of cell types in each sample from ECC patients delineated by single-cell transcriptomic analysis.** (**A**) Experimental workflow of scRNA-seq procedure for ECC tumors and paratumor tissues. (**B**) Hematoxylin and eosin (HE) staining on paratumor and tumor slides of the 5 ECC patients. Scale bars, 100 mm. (**C**) Samples obtained from 5 EC patients and clinicopathological characteristics of the 5 patients, more details are provided in Supplementary Figure 1B. (**D**) The remaining cell fraction (left bar plot) and cell number (right bar plot) after quality control and filtering step for each biopsy. (**E**) The t-distributed stochastic neighbor embedding (t-SNE) plot demonstrates the major cell types in each sample. (**F**) Expression of representative marker genes of the major cell types defined in EC samples. (**G**) The percentage of cell types assigned to each sample in (**E**). Pie charts of cell-type fractions for tumor-infiltrating immune cells of each patient, colored by cell type.

Focus was put on endometrioid carcinomas to explore the cellular diversity in ECC because they are the most common type of EC [5]. 20,008 cells collected from 5 primary endometrial carcinomas were profiled using the single-cell RNA-seq. For comparison, 11,510 cells from the paratumor tissues (1 cm away from the tumor boundary, Supplementary Figure 1A) of three patients were also profiled (Figure 1A–1D). For patients EC4 and EC5, the tumor sizes were too big to acquire the paired paratumor tissues. The five patients ranged from 42 to 68 years old, with grade 1, FIGO stage I tumors (except for EC5 with FIGO stage II tumor) and without lymph node metastasis. In all patients, PTEN expression was negative, POLE display was intact, and the mismatch repair (MMR) proteins expression was positive. However, for EC5, one of the MMR proteins (MSH6) expression was negative (Figure 1B, 1C and Supplementary Figure 1B). Paratumor and tumor samples were obtained after resection and immediately processed into single-cell suspensions of enriched viable cells. The corresponding cell populations were then subjected to 3′ mRNA single-cell transcriptome analysis (scRNA-seq) using the 10x Genomics Chromium platform (Figure 1A). The average mean reads per cell was 199,328, and the average median number of genes detected per cell was 2,571 (Supplementary Figure 2A). After quality filtering using the seurat package, 19,505 cells from tumor samples and 11,275 cells from paratumor samples remained for downstream analysis (Figure 1D). Graph-based clustering of the informative principle components (n = 20) was then done to classify cellular compositions. Known marker genes were then used to identify the major cell types such as epithelial cells, endothelial cells, fibroblasts, T cells, B cells and myeloid cells in each sample (Figure 1E, 1F). Variations in the number of cell type composition across tumors were observed (Figure 1G and Supplementary Figure 2B). For example, the fibroblasts fractions constituted 46% and 22% in EC1-P and EC1-T respectively and only less than five percent (< 5%) in other samples (Figure 1G and Supplementary Figure 2B). Interestingly, we found that p16 was expressed in the spindle-shaped stromal cells while not commonly in glandular epithelial cells in patient EC3 (Supplementary Figure 1B, 1C). p16 is commonly used as a biomarker for diagnosing gynecological malignancies [15]. Differences in the p16 expression status varied according to the degree of malignancy and histological type [16]. Endometrioid carcinomas usually show patchy p16 expression on the glandular epithelium, while stromal p16 expression is uncommon [17]. The spindle-shaped stromal cells are mainly fibroblasts, and the higher fibroblasts fraction of patient EC3 might have resulted from uncommon p16 expression. For patient EC4, the big tumor size and a high Ki67 index of 30% may be related to its low immune cell fraction and high epithelial cell fraction (Supplementary Figure 1B, 1C). For patient EC5, one of the mismatch repair (MMR) proteins — MSH6 expression was negative, suggesting an MSI type (Supplementary Figure 1B, 1C). Both MSI-positive endometrial cancers and POLE-mutated endometrial cancers have high neoantigen loads and immunogenic phenotypes [18, 19], which may explain the high immune cell fraction in EC5.

**Figure 2:**
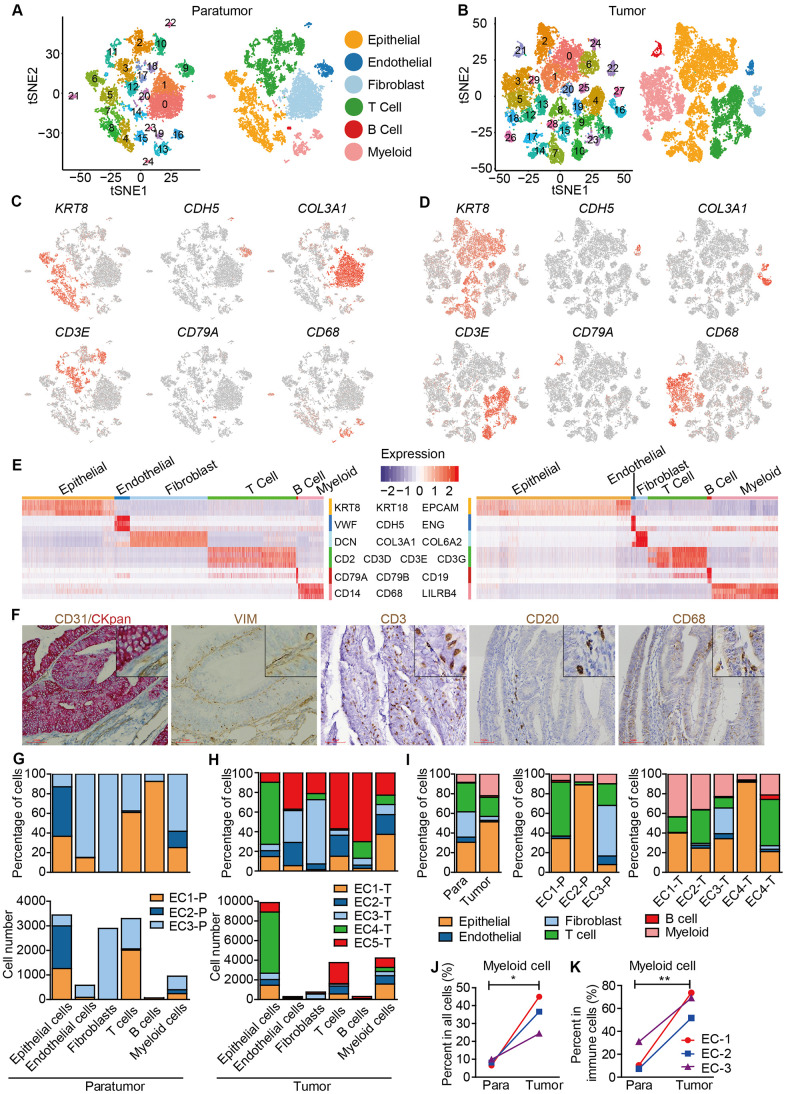
**Unbiased characterization of multiple cell types from integrated tumor and paratumor samples.** (**A**, **B**) t-SNE projection of the 11,275 cells from 3 integrated paratumor samples (**A**) and 19,505 cells from 5 integrated tumor samples (**B**), color-coded by their associated cluster (left) or the assigned cell type (right). (**C**, **D**) Expression of marker genes of each cell type defined in A and B. (**E**) Heatmap showing the expression levels of known markers of epithelial cells epithelial cells (KRT8, KRT18, EPCAM), endothelial cells (CDH5, VWF, ENG), fibroblasts (COL3A1, COL6A2, DCN), T cells (CD2, CD3D, CD3E, CD3G), B cells (CD79A, CD79B, CD19) and myeloid cells (CD14, CD68, LILRB4) in Paratumor (left panel) and Tumor (right panel). (**F**) Representative images of epithelial cells, endothelial cells, fibroblasts, T cells, B cells and myeloid cells of EC4, after IHC staining with CKpan, CD31, VIM, CD3, CD20 and CD68 antibodies, respectively. Scale bars, 80 mm. (**G**, **H**) For each cell type: the cell fractions and numbers originating from each of the 3 paratumor and 5 tumor samples are shown. (**I**) The fractions of the six cell types in paratumor and tumor samples (left), and in each patient (middle and right). (**J**, **K**) The percentages of myeloid cells of all cells (**J**) and immune cells (**K**) in paratumor and tumor samples. Data were analyzed using a students’ t-test, *P < 0.05, **P < 0.01.

Data from 3 paratumor samples and 5 tumor samples was merged respectively to enable systematic comparison across patients. The merged data was then used to performed principle component analysis. PCA analysis revealed that there were 25 clusters in paratumor and 30 clusters in tumor datasets (Figure 2A, 2B and Supplementary Figure 3A, 3B). The clusters were annotated by the expression of known marker genes as epithelial cells (*KRT8*, *KRT18*, *EPCAM*), endothelial cells (*CDH5*, *VWF*, *ENG*), fibroblasts (*COL3A1*, *COL6A2*, *DCN*), T cells (*CD2*, *CD3D*, *CD3E*, *CD3G*), B cells (*CD79A*, *CD79B*, *CD19*) and myeloid cells (*CD14*, *CD68*, *LILRB4*) (Figure 2A–2E). Immunohistochemistry (IHC) staining also performed to confirm the existence of different cell types (Figure 2F). Variations in the fraction of different cell types across tumors were observed (Figure 2G, 2H). Cell-to-cell correlations constructed from gene expression of the stromal single cells revealed separation of cells by cell-types while not by sample origin, suggesting that most stromal cell type populations were shared by different patients (Supplementary Figure 3C) [20]. Epithelial cells, T cells and myeloid cells were present in all patients, while the fibroblasts and endothelial cells showed relatively low fractions (Figure 2I). The low fractions of fibroblasts and endothelial cells may result from their well-known poor dissociation efficiency following tissue disaggregation [12], with fibroblasts and endothelial cells being more embedded in extracellular matrix and basement membrane than immune cells, and hence more difficult to dissociate. The percentage of myeloid cells was increased significantly in tumor samples (Figure 2J, 2K). Enrichment of myeloid cells in tumor samples was also improved by IHC staining of the ECC tumor and paratumor sections with CD68 antibody (Figure 4C, 4D).

### Macrophages were strongly enriched in the tumor and show a continuous range of macrophage activation states

Lymphoid and myeloid cells are immune cells that are clinically impactful while the ECC malignant cells originate from endometrial epithelial cells [21]. As such, these three major cell types were further analyzed in-depth by identifying sub-clusters within each of them.

**Figure 3:**
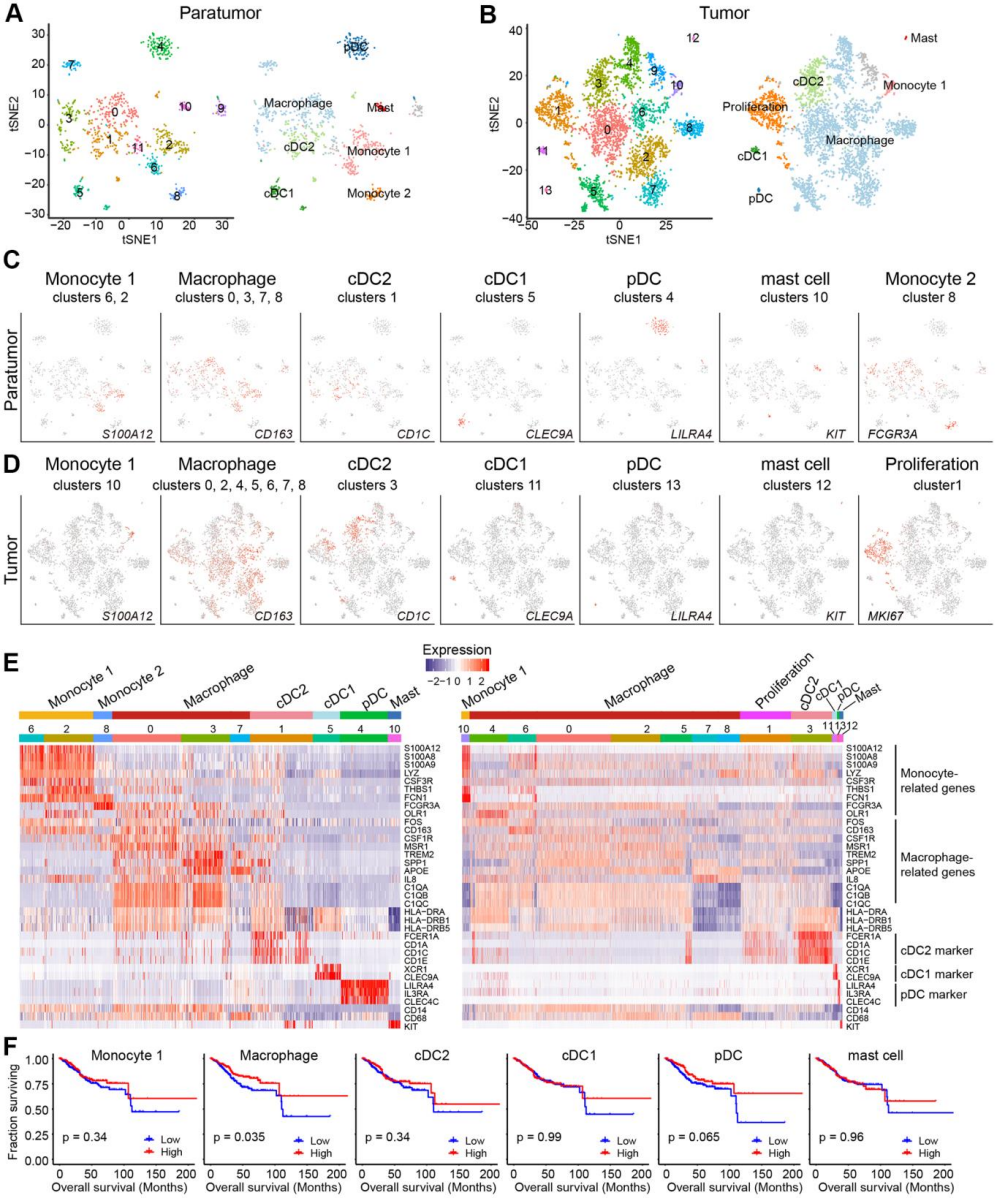
**Myeloid cell clusters in paratumors and endometrial tumors.** (**A**, **B**) t-SNE plot of 936 myeloid cells in Paratumor (**A**) and 4,152 myeloid cells in Tumor (**B**), color-coded by their associated cluster (left) or the assigned subtype (right). (**C**, **D**) t-SNE plot, color-coded for relative expression (lowest expression to highest expression, gray to red) of marker genes for the myeloid subtypes in Paratumor (**C**) and Tumor (**D**). (**E**) Heatmaps created using known gene expression profiles of myeloid cells in Paratumor (left panel) and Tumor (right panel). The identity of each cluster was assigned using known markers. (**F**) The overall survival curves based on TCGA-UCEC data (n = 549 patients), stratified for the average expression (binary: high versus low) of tumor myeloid cell marker genes.

**Figure 4:**
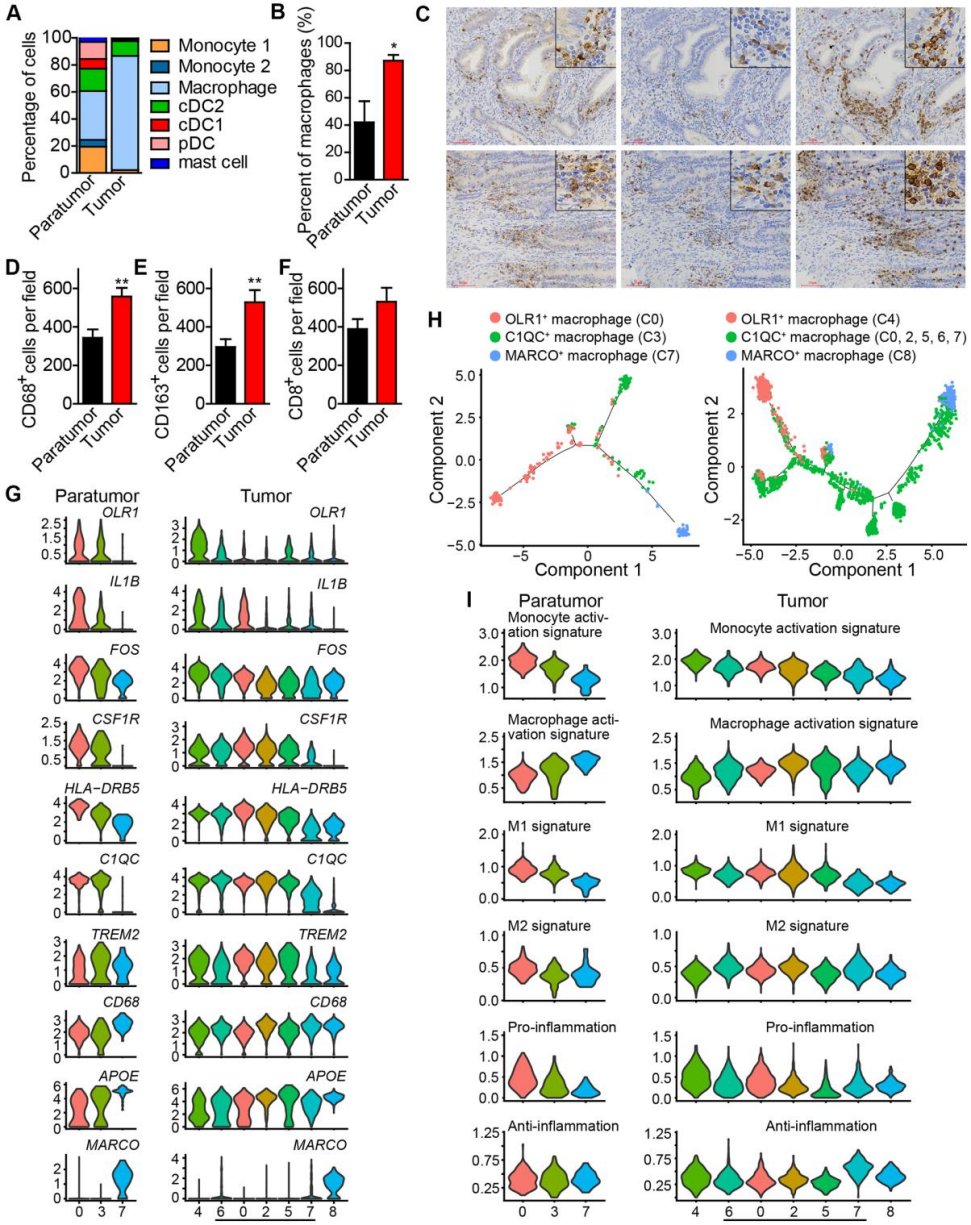
**Enrichment of macrophages in endometrial tumors and distinct macrophage populations.** (**A**) The fractions of myeloid subtypes in Paratumor and Tumor. (**B**) The percentage of macrophages in paratumor and tumor tissues. *P < 0.05, Student’s t test. (**C**) IHC staining images of CD68, CD163 and CD8 in paratumor and tumor slides isolated from endometrial carcinoma sections. Scale bars, 80 μm. (**D**–**F**) Quantification of the numbers of CD68^+^, CD163^+^ and CD8^+^ cells as presented in (**C**) Data are means ± SEM (30 paratumor sections and 40 tumor sections were analyzed). **P <0.01 versus paratumor group (Student’s t-test). (**G**) Violin plots displaying the expression profile of representative genes related to monocyte-macrophage lineage across the macrophage clusters in Paratumor (left panel) and Tumor (right panel). The y axis shows the normalized expression. (**H**) Pseudo-time analysis of 3 macrophage populations from Paratumor (left) and Tumor (right) inferred by Monocle2. Each point corresponds to a single cell, and each color represents a macrophage population as indicated. (**I**) Violin plots indicating relative expression levels of monocyte activation, macrophage activation, M1, M2, pro-inflammation and anti-inflammation gene signatures across the macrophage clusters in Paratumor (left panels) and Tumor (right panels).

After re-clustering, 878 myeloid cells in 10 clusters and 3,966 myeloid cells in 13 clusters were detected in paratumor and tumor samples respectively (Figure 3A, 3B and Supplementary Figure 4A, 4B). Within the myeloid cells, four distinct meta-subsets: monocytes, macrophages, dendritic cells (DCs) and mast cells were also identified. Overall, the percentage composition of monocytes, dendritic cells and mast cells were higher in Paratumor than in Tumor i.e. 24.5% (215 of 878) vs 2.2% (86 of 3,966), 36.2% (318 of 878) vs 12.7% (504 of 3,966) and 3.1% (27 of 878) vs 0.6% (24 of 3,966), respectively. On the other hand, the percentage composition of macrophages was higher in Tumor than in Paratumor i.e. 84.5% (3352 of 3,966) vs 36.2% (318 of 878), respectively (Figure 4A and Supplementary Figure 4C–4E). 2 subtypes in monocytes were further analyzed: CD14^+^S100A12^+^ population 1 [22] (cluster 2, 6 in Paratumor, and cluster 10 in Tumor) was transcriptionally similar to “classical” monocytes, and FCGR3A^+^ population 2 (cluster 8 in Paratumor) was similar to “nonclassical” monocytes (Figure 3C–3E) [23]. DCs were further subdivided into cDC1 (cross-presenting dendritic cells; cluster 5 in Paratumor and cluster 11 in Tumor; *CLEC9A*^+^ and *XCR1*^+^), cDC2 (cluster 1 in Paratumor and cluster 1 in Tumor; *CD1C*^+^) and plasmacytoid DC (pDC; cluster 4 in Paratumor and cluster 13 in Tumor; *LILRA4*^+^ and *IL3RA*^+^) (Figure 3C–3E). Three clusters (0, 3 and 7 in Paratumor) corresponded to paratumor macrophages while seven clusters (0, 2, 4, 5, 6, 7 and 8 in Tumor) corresponded to tumor macrophages (Figure 3C–3E). ECC infiltrating myeloid subtypes mostly consisted of cells from three or more patients (Supplementary Figure 4D). We evaluated whether the relative presence of ECC infiltrating myeloid subtypes impacts patient survival using TCGA-UCEC data. We found that tumor infiltrating macrophages were associated with increased overall survival (Figure 3F). The macrophages showed strongly enrichment in the tumor samples (Figure 4B). Similarly, their enrichment was also improved by IHC staining of EC tumor and paratumor sections with CD163 antibody (Figure 4C, 4E), while no significant difference was showed by CD8 staining (Figure 4C, 4F). This was an indication that macrophages exert an important effect on tumor behavior.

Based on these findings, further analysis of the macrophages was done. Three distinct populations in macrophages; OLR1^+^ macrophage population 1 (cluster 0 in Paratumor, and cluster 4 in Tumor) [22], C1QC^+^ macrophage population 2 (cluster 3 in Paratumor, and cluster 0, 2, 5, 6, 7 in Tumor) [24], and MARCO^+^ macrophage population 3 (cluster 7 in Paratumor, and cluster 8 in Tumor) [25, 26] were observed (Figure 4G). As the cell states from population 1, 2 to 3, key macrophage-associated genes, such as APOE and complement genes (C1QA, C1QB, and C1QC) were broadly expressed across clusters in continuous gradients. The expression of genes such as CD14, FOS, HLA-DRB5, and IL-1B decreased, while the expression of CD68 and APOE increased (Figure 4G). The Monocle 2 algorithm was employed to characterize macrophages. Results indicated that macrophages comport with a continuous activation model that began with the OLR1^+^ macrophages, followed by C1QC^+^ macrophages and ended with MARCO^+^ macrophages (Figure 4H). OLR1^+^ early activated macrophages were enriched with monocyte activation genes whereas MARCO^+^ macrophages were enriched with macrophage activation genes (Figure 4I). Profiling of macrophages in terms of M1 and M2 signatures revealed that activation of macrophages was negatively correlated with M1 level but there was no association with M2 level (Figure 4I). Characterization of macrophages in terms of pro-inflammatory and anti-inflammatory signatures showed that activation of macrophages was also negatively correlated with enrichment of pro-inflammatory factors but it was not associated with the level of anti-inflammatory factors (Figure 4I). These results support the idea that macrophage activation in the tumor microenvironment does not comport with the polarization model wherein M1 and M2 activation states exist as mutually exclusive discrete states, consistent with the study by Azizi et al in 2018 [26]. These formed the basis for further examination into the role of monocyte/macrophage subsets in endometrial carcinomas.

### Tumor T cells downregulate immune activation pathways and tumor CD8^+^ T cells show higher exhaustion level

**Figure 5:**
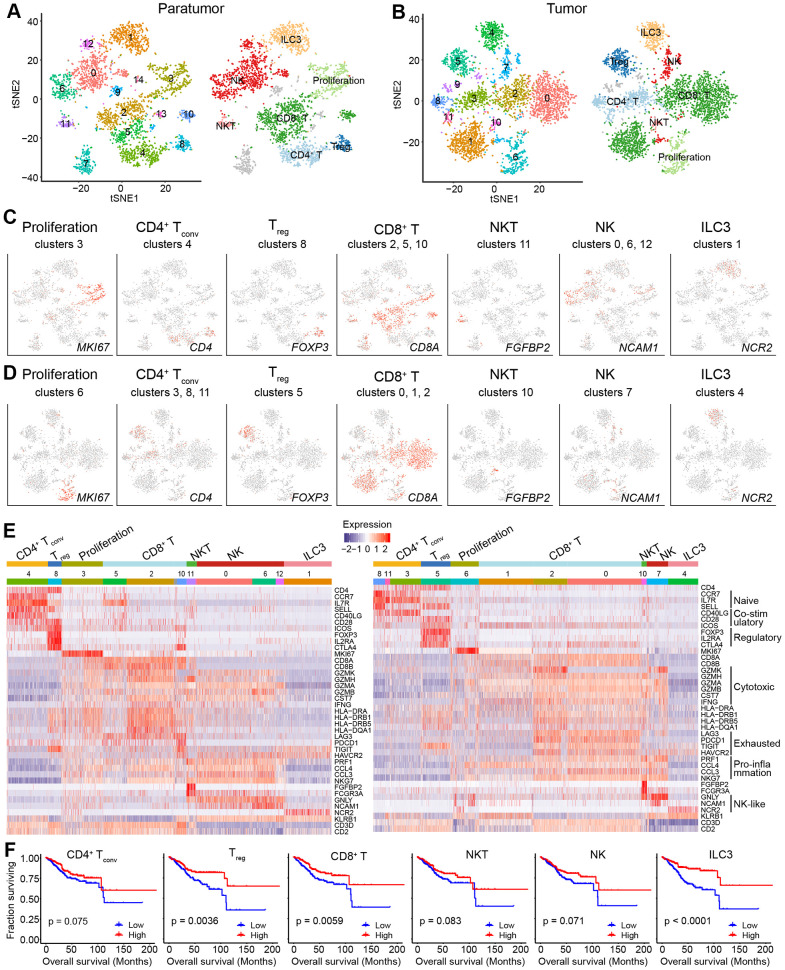
**Lymphoid cell clusters in paratumors and endometrial tumors.** (**A**, **B**) t-SNE plot of 3,223 lymphoid cells in Paratumor (**A**) and 3,712 lymphoid cells in Tumor (**B**), color-coded according to their associated cluster (left) or the assigned subtype (right). (**C**, **D**) t-SNE plot, color-coded to show the relative expression (gray to red) of marker genes for the lymphoid subtypes in Paratumor (**C**) and Tumor (**D**). (**E**) Heatmap created using known gene expression profiles of lymphoid cells in Paratumor (left panel) and Tumor (right panel). The gene expression profiles include marker genes for cell type and naive, costimulatory, regulatory, exhaustion and cytotoxicity expression signatures. The identity of each cluster was assigned with known markers. (**F**) The overall survival curves based on TCGA-UCEC data (n = 549 patients), stratified for the average expression (binary: high versus low) of tumor lymphoid cell marker genes.

**Figure 6:**
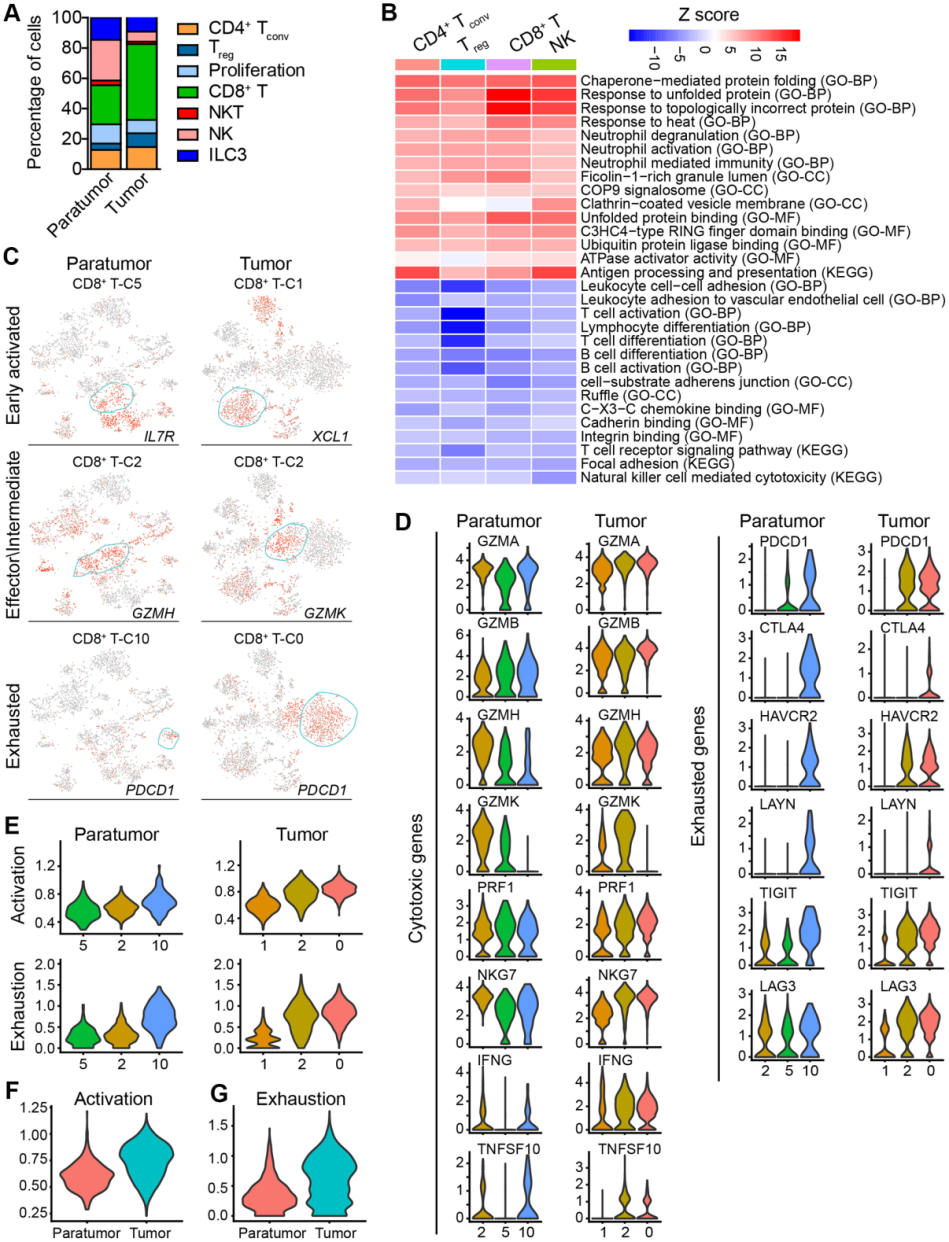
**Subtype marker genes expressed in lymphoid cells and functional genes expressed in CD8^+^ T cells.** (**A**) The fractions of lymphoid subtypes in Paratumor and Tumor. (**B**) Enriched pathway activities for up- (red) and down- (blue) regulated genes, between T cells from Tumor versus Paratumor. Color key from white to red indicates z-score of -Log_10_(*P* value), color key from white to blue indicates z-score of Log_10_(*P* value). (**C**) t-SNE plot, color-coded to show the relative expression (gray to red) of marker genes for the 3 states of indicated CD8^+^ T cells. (**D**) Violin plots displaying the expression profile of cytotoxic and exhausted genes of the CD8^+^ T cell clusters in Paratumor (left panel) and Tumor (right panel). (**E**) Violin plots indicating relative expression levels of T cell activation and exhaustion gene signatures across the CD8^+^ T cell clusters in Paratumor (left panel) and Tumor (right panel). (**F**, **G**) Violin plots indicating relative expression levels of T cell activation (**F**) and exhaustion (**G**) gene signatures in different tissue types.

Tumor-infiltrating lymphocytes (TILs) such as CD8^+^ T cells are essential for successful immune surveillance and tumor killing. In this study, gene expression profiles of distinct lymphocyte populations were examined to yield a comprehensive understanding of the ECC TIL landscape. Through the scRNA-seq data, 2,896 lymphocytes in 11 clusters and 3,591 lymphocytes in 11 clusters were detected in paratumor and tumor samples, respectively (Figure 5A, 5B and Supplementary Figure 5A). The lymphocyte repertoire was categorized into two broad groups: conventional T cells and innate lymphoid cells (Supplementary Figure 5B). Conventional T cells included conventional CD4^+^ T cells (cluster 4 in Paratumor and cluster 3, 8, 11 in Tumor; *CD4^+^*), regulatory T cells (cluster 8 in Paratumor, and cluster 5 in Tumor; *FOXP3^+^*), CD8^+^ T cells (cluster 2, 5, 10 in Paratumor, and cluster 0, 1, 2 in Tumor; *CD8A^+^*) (Figure 5C–5E and Supplementary Figure 5C). Innate-like lymphoid cells included natural killer T cells (NKT cells) (cluster 11 in Paratumor, and cluster 10 in Tumor; *FGFBP2^+^* and *FCGR3A*^+^) [12], natural killer cells (cluster 0, 6, 12 in Paratumor, and cluster 7 in Tumor; *NCAM1*^+^ and *GNLY*^+^) and type 3 innate lymphoid cells (ILC3) (cluster 1 in Paratumor, and cluster 4 in Tumor; *NCR2*^+^) (Figure 5C–5E and Supplementary Figure 5C). Cluster 3 in Paratumor and Cluster 6 in Tumor had proliferating cells (*MKI67*^+^) of various lymphocyte lineages (Figure 5C–5E and Supplementary Figure 5C). Although the number of EC infiltrating lymphocytes showed variation across both the tissues and patients, ECC infiltrating lymphocyte subtypes mostly consisted of cells from three or more patients (Figure 6A and Supplementary Figure 5D–5F). We evaluated whether the relative presence of ECC infiltrating lymphocyte subtypes impacts patient survival using TCGA-UCEC data. We found that 3 tumor infiltrating lymphocyte subtypes (CD8^+^ T cells, regulatory T cells and type 3 innate lymphoid cells) were associated with increased overall survival (Figure 5F). A comparison of the pathway expression levels between paratumor and tumor T cells revealed pervasive changes. The expression levels were mostly coherent across four T-cell types detected. The T-cell types had an increased response to unfolded protein and neutrophil activation, while decreased lymphocyte differentiation and activation in tumor samples (Figure 6B and Additional File 1).

Moreover, gene expression programs for distinct cell functional status in T cells were analyzed. These expression programs included naive, costimulatory, regulatory, exhaustion and cytotoxicity expression signatures (Figure 5E). CD8^+^ T cells could be further identified into four distinct cell states. Early activated (pro-memory) CD8^+^ T cells expressed marker genes IL7R [27, 28] (cluster 5 in Paratumor) or XCL1 [29] (cluster 1 in Tumor) while with low expression of activated makers such as HLA-DR (Figures 5E and 6C). Effector memory CD8^+^ T cells (cluster 2 in Paratumor) were characterized by the expression of GZMH and other activated cytotoxic genes associated with effector functions (Figures 5E, 6C and 6D). GZMK^+^ CD8^+^ T cells [30] (cluster 2 in Tumor) represented an intermediate state between the effector and exhausted T cells. They showed high expression of activated makers like effector cells, while also shared a few common genes with exhausted cells such as PDCD1 (Figures 5E, 6C and 6D). Exhausted (terminal differentiation) CD8^+^ T cells (cluster 10 in Paratumor and cluster 0 in Tumor) were enriched with exhaustion genes CTLA4, PDCD1 and HAVCR2 (Figures 5E, 6C and 6D). Evaluation of the expression of T cell activation and exhaustion genes of distinct populations revealed that the exhaustion program was relative to that of activation genes (Figure 6E). This observation was consistent with the “activation-dependent exhaustion expression program” reported previously [31, 32]. A comparison of the expression of exhaustion gene sets of overall paratumor and tumor CD8^+^ T cells showed that tumor cells display higher “exhaustion scores” (Figure 6F, 6G). These results provided a baseline description of the lymphocyte transcriptomes in endometrial carcinomas.

### Endometrial epithelial cells

The human endometrium forms the uterine cavity. It is a highly regenerative organ that undergoes menstrual cycle in response to the ovarian hormones, estrogen and progesterone [33, 34]. After puberty, the endometrium is functionally divided into two major zones, the basalis and the functionalis. The functionalis is composed of luminal epithelium, loose stroma and the superficial glands. It is shed at menses. On the other hand, the basalis resides at the endometrial/myometrial interface and contains compact stroma and the deeper glands. It is the source for the regeneration of the functionalis layer [33]. The endometrial epithelial cells consist of secretory cells and ciliated cells. It is also proposed that there exist progenitor/stem cells that reside in the basalis layer [35].

**Figure 7:**
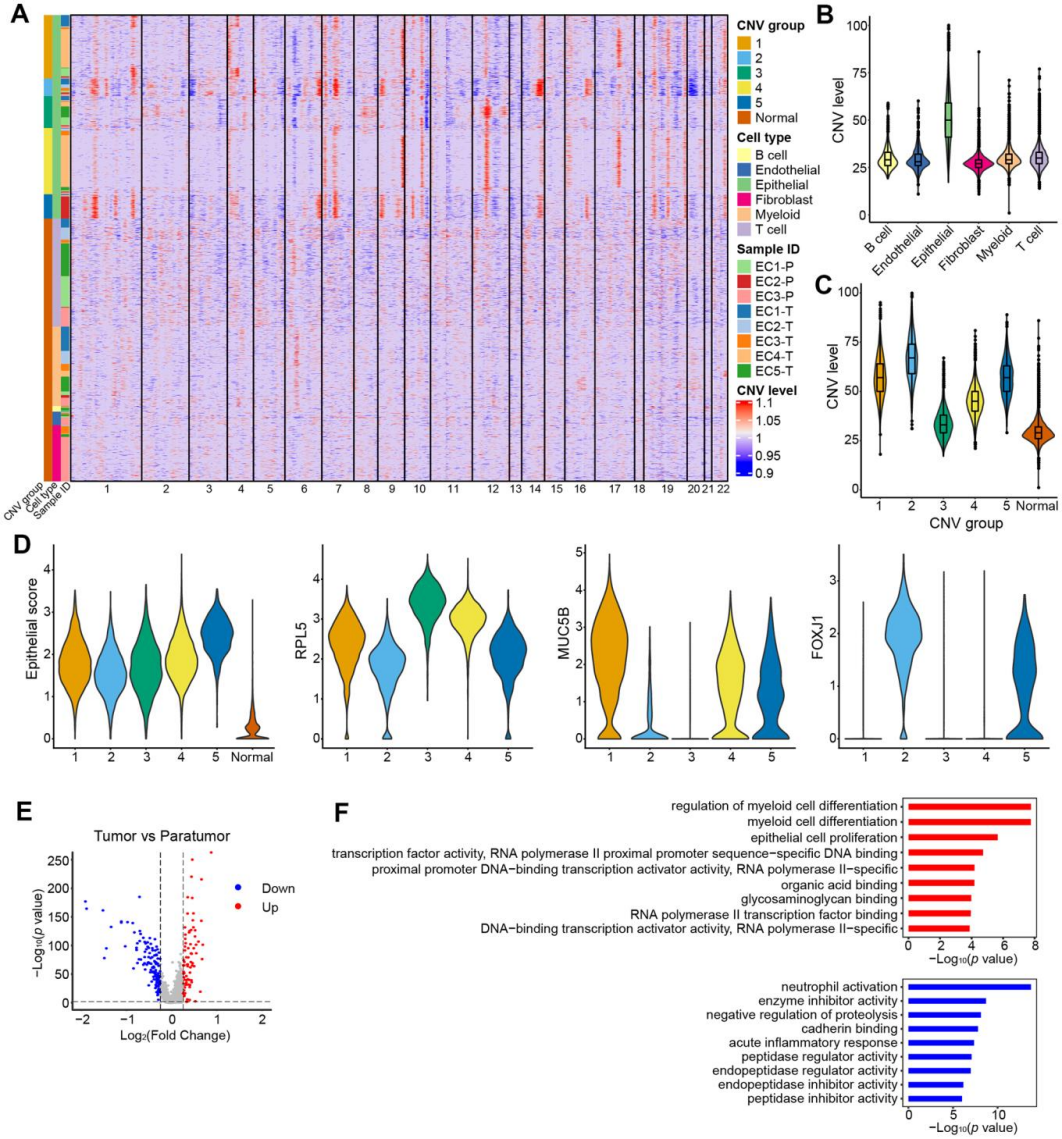
**CNV landscape of epithelial cells.** (**A**) Heatmap showing large-scale CNVs for individual cells (rows) of eight samples from five patients. (**B**) Violin plots showing distributions of CNV scores among different cell types. (**C**) Violin plots showing distributions of CNV scores among different CNV groups. (**D**) Violin plots showing expression of epithelial subtype-related genes among different CNV groups. (**E**) Differential expressed genes detected between tumor samples vs. their paratumor counterparts from EC1-3. (**F**) Representative enriched GO terms for up- (red) and down- (blue) regulated genes as displayed in E, respectively.

**Figure 8:**
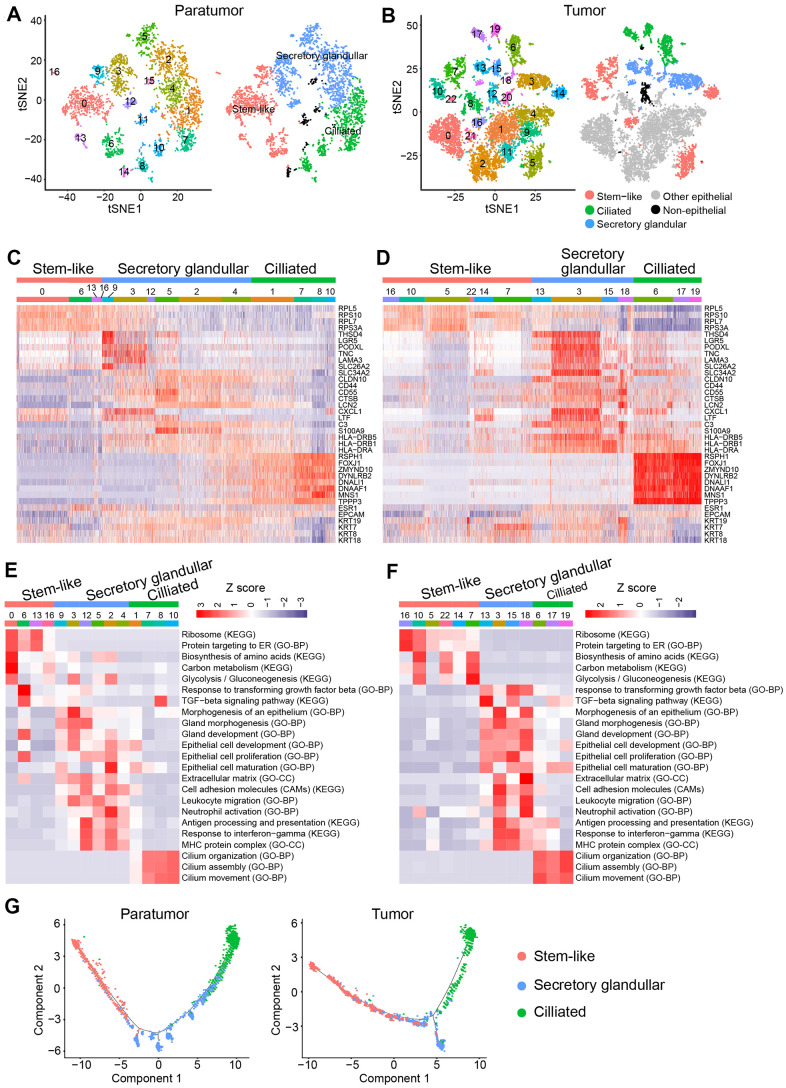
**Endometrial epithelial cell clusters in paratumors and endometrial tumors.** (**A**, **B**) t-SNE plot of 3,403 endometrial epithelial cells in Paratumor (**A**) and 9,789 endometrial epithelial cells in Tumor (**B**), color-coded by their associated cluster (left) or the assigned subtype (right). (**C**, **D**) Heatmap analysis using known gene expression profiles of endometrial epithelial cells from Paratumor (**C**) and Tumor (**D**). The identity of each cluster was assigned by known markers recently reported. (**E**, **F**) The enriched gene ontology terms for genes with specific expression in each endometrial epithelial cluster in Paratumor (**E**) and Tumor (**F**). Color key from blue to red indicates z-score of -Log_10_(*P* value). (**G**) Pseudo-time analysis of endometrial epithelial cells from Paratumor (left) and Tumor (right) inferred by Monocle2. Each point corresponds to a single cell, and each color represents an endometrial epithelial subtype as indicated.

Endometrial carcinoma arises from epithelial cells. Therefore, presumably the epithelial compartment contains the malignantly transformed tumor cells. To distinguish the malignant status of cells, we calculated large-scale chromosomal copy number variation (CNV) for each cell type based on the average expression patterns across intervals of the genome (See Materials and Methods). We found that epithelial cells exhibited remarkably higher CNV levels than other types of cells (Figure 7A, 7B). The epithelial cells were further divided into five groups (Figure 7A, 7C). All five groups showed high expression of epithelial genes (Figure 7D). Specifically, group 3 showed a low CNV level and high expression of ribosomal genes such as RPL5 which are enriched in stem-like subtype; group 2 showed a high CNV level and high expression of ciliated cell-associated genes such as FOXLJ1; group 1 showed a moderate CNV level and high expression of secretory glandular-associated genes such as MUC5B; group 4 showed an intermediate status between G3 and G1 with both a moderate expression of RPL5 and MUC5B; group 5 showed an intermediate status between G1 and G2 with both a moderate expression of FOXLJ1 and MUC5B (Figures 7D, 8C and 8D). The CNV level related to epithelial cell subtypes was consistent with the developmental trajectory revealed by Pseudo-time analysis (Figure 8G). Gene expression differences were compared in the epithelial cells of the tumor samples vs. their paratumor counterparts in EC1-3. We identified 227 differentially expressed genes (Figure 7E and Additional File 2). The gene set enrichment analyses showed that genes up-regulated in tumor samples were mainly enriched for cancer-related functions, such as epithelial cell proliferation and enhanced RNA polymerase II (Pol II) function, suggesting the malignant state (Figure 7F). In contrast, genes expressed at higher levels in the paratumor counterparts were mainly related to negative regulation of proteolysis, and its enzymes—endopeptidase and peptidase activity (Figure 7F). Proteolytic enzymes are active in the tumor microenvironment, and are believed to be related to tumor progression [36, 37]. Therefore, the inhibition of endopeptidase and peptidase activity in paratumor tissues is well understood.

Further analysis was performed to better understand the cell subtypes in epithelial cells. 3,403 and 9,789 epithelial cells in paratumor and tumor samples respectively were profiled. The t-SNE projection revealed that there were 17 clusters in Paratumor and 23 clusters in Tumor (Figure 8A, 8B). Examination of gene expression patterns and gene ontology enrichment analysis were done to identify the cluster characteristics and to study the cluster function in each cluster (Figure 8C–8F). The cell clusters were then assigned to three epithelial subtypes using known markers obtained from the published literatures [35, 38, 39] (Figure 8C, 8D). However, a large number of cells in tumor samples could not be assigned to any of the three epithelial subtypes. Majority of these clusters originated from one patient (EC4) indicating that their marker genes were patient specific (Figure 8B). Stem-like cells, secretory glandular cells and ciliated cells were identified in both paratumor and tumor samples (Figure 8C, 8D). Ciliated cells expressed high levels of markers such as the ciliated marker TPPP3, the radial spoke gene RSPH1 and the dynein assembly genes DNAAF1 and ZMYND10 related to motile cilia and ciliogenesis as well as transcription factor FOXJ1 (Figure 8C, 8D). Gene ontology analysis of the cluster specific genes further revealed terms related to cilia organization, assembly and movement for the ciliated subtype (Figure 8E, 8F). Secretory glandular cells highly expressed genes related to epithelial cell development and differentiation (PODXL, LGR5, CLDN10, SLC26A2 and S100A9) and gland morphogenesis (TNC and LAMA3) (Figure 8C, 8D). Other than epithelial cell development and gland development, gene ontology analysis of secretory glandular cell cluster also showed terms related to extracellular matrix, cell adhesion molecules and leukocyte migration indicating that they might interact with surrounding stromal cells (Figure 8E, 8F). Stem-like cells did not express specific marker genes but showed elevated expression of ribosomal genes indicating that there was presence of stem/progenitor cells (Figure 8C, 8D) [40, 41]. The Monocle 2 algorithm was performed on the three epithelial subtypes to establish their developmental trajectories. Pseudo-time analysis revealed that the different subtypes were formed in a relative developmental trajectory that began with the stem-like cells, followed by secretory glandular cells and ended with ciliated cells. This indicated that there was a possibility that stem-like cells could transform to secretory glandular cells and then to ciliated cells (Figure 8G). This result is similar to the mucociliary differentiation trajectory of nasal epithelial cultures published by Ruiz Garcia et al. [42]. These results extended the human endometrial epithelial transcriptional signature.

## DISCUSSION

The host immune system can play paradoxical roles in tumor outgrowth. On one hand, tumor infiltrating lymphocytes (TILs) especially the CD8^+^ T cells are essential for tumor killing. On the other hand, myeloid compartment especially the macrophages exert tumor-promoting activities [43]. Despite the development of cancer immunotherapies such as immune checkpoint inhibitors, mechanisms of drug response or prediction of efficacy remain elusive. This is because of the heterogeneous immune composition in tumors. In this study, unbiased single-cell RNA-seq analysis was used to construct an immune atlas of endometrial carcinomas by combining immune cells isolated from tumor and paratumor tissues. This atlas revealed cellular diversities of both the lymphoid and myeloid compartment.

In this study, CD8^+^ T cells in endometrial carcinomas displayed a continuous spectrum of T cell activation states of early activated, effector memory, intermediate state between effector and exhausted, and exhausted. Exhausted CD8^+^ T cells were accumulated in tumor samples. However, immune checkpoint blockade of PD1/PDL-1 pathway was shown to reinvigorate exhausted CD8^+^ T cells with intermediate expression of PDCD1, but not those with high PDCD1 expression [44]. This meant that it was more beneficial if T cell exhaustion could be reversed earlier rather than later. GZMK cluster expressed both cytotoxic and exhausted genes possibly representing a transition state between effector and exhausted T cells [30]. As such, promoting transition of GZMK^+^ CD8^+^ T cells back to effector-like cells and preventing them from further exhaustion would be a strategy for cancer immunotherapy.

Ongoing studies revealed pro-tumor role of macrophages in endometrial carcinomas [45]. In this study, three macrophage populations were discovered that had a continuous range of macrophage activation states. Activated macrophage populations 2 and 3 were enriched in the tumor tissues and had lower expression of M1 phenotype signature compared to the OLR1^+^ macrophages. Activated macrophage populations 2 and 3 lowly expressed IL-1B suggesting that it had an anti-inflammatory property. Based on these findings, switching the macrophage transcriptome towards an M1 phenotype could be a potential EC therapeutic strategy. MARCO was reported to be associated with M2 phenotype [26] and was linked to poorer outcomes in human breast cancer [46]. Another study found that MARCO-positive macrophages secreted less TNF-α in response to LPS/ IFN-γ stimulation than MARCO-negative CD68^+^ macrophages [25]. Therefore, the subpopulation of macrophages which expressed MARCO might serve as a new immunotherapy target.

Characterization of endometrial epithelial cell subtypes is problematic because of the lack of specific markers for isolating and examining their functional properties. In this study, features of gene expression profiles of three identifiable endometrial epithelial cell subtypes were characterized through gene expression patterns examination and gene ontology analysis based on scRNA-seq data. Progenitor/stem cells are postulated to reside in the basalis layer and are the source of regrowth of the functionalis layer. However, the two major zones of the endometrium are not anatomically partitioned. Studies on endometrial epithelial cell types that exist in different regions of the endometrium are sparse. Because spatial information of cells in the tissue is often lost during the single-cell suspension preparation steps, direct correlation of the subtypes with endometrium regions/layers is essential. As such, new techniques such as single-molecule FISH (smFISH), laser capture microdissection and laser scanning microscopy are required [11]. These techniques were not a part of this study and thus the layer property of the cell subtypes requires more careful examination. In tissues that renew rapidly such as epithelium, skin and gastrointestinal tract, stem cells and their progeny in the epithelial lineage are responsible for tumor initiation because they have a long life span that allow accumulation of genetic damage [33, 47]. Currently, there are no credible specific epithelial stem cell surface markers [48] thus necessitating further investigations to answer whether there are stem cells in the stem-like cell subtype.

**Figure 9:**
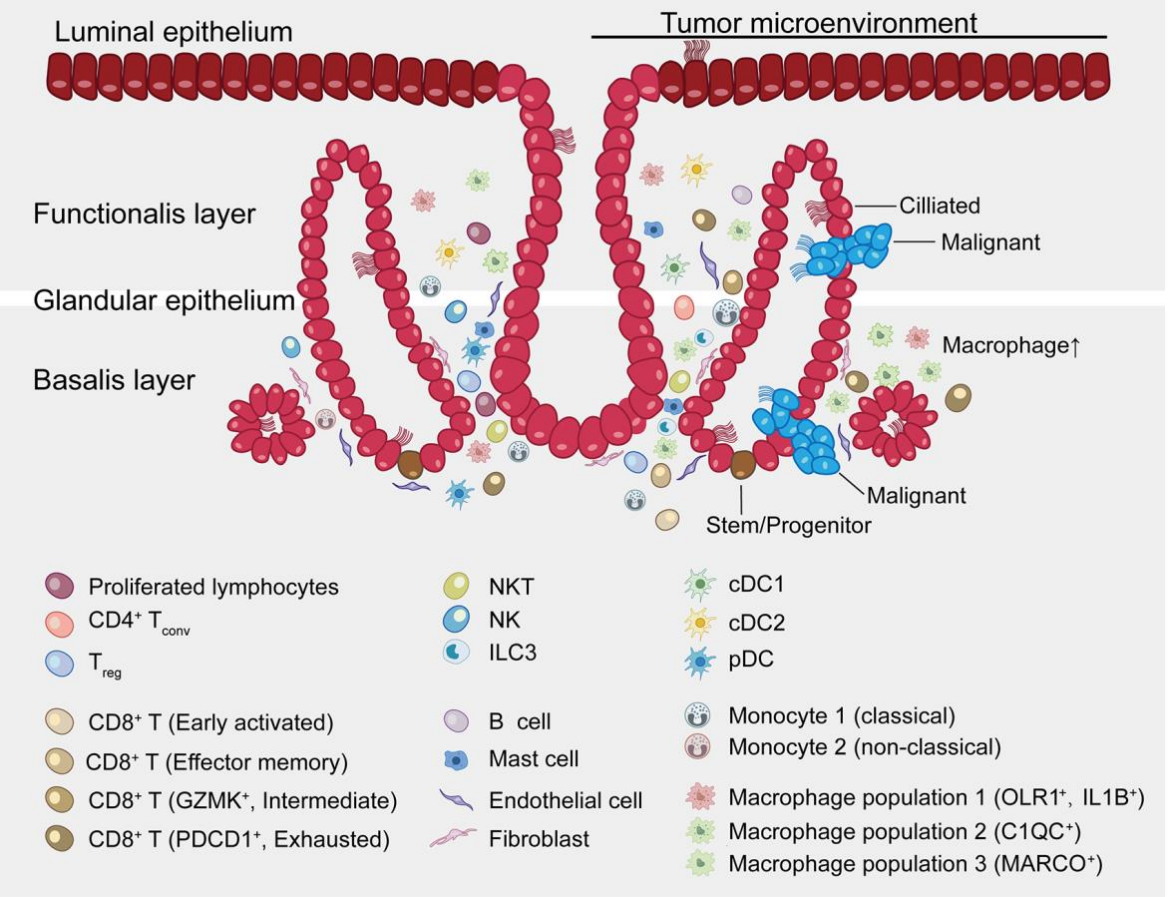
**Summary map of the endometrial carcinoma ecosystem.** The endometrium is the inner lining of the uterus and consists of epithelial and stromal cells. These are further divided into glandular (GE) and luminal (LE) epithelium. The basalis layer is the source for the regeneration of the endometrium. The two major zones of the endometrium, the basalis and the functionalis, are functionally divided while not anatomically partitioned. The subtype of epithelial cells was not confirmed by immunohistochemical staining and is inferred as a result of pathway analysis and transcriptional similarity to the published gene expression patterns. Stromal cells consist of endothelial cells, fibroblasts, myeloid cells- including DCs, monocytes, macrophages, mast cells, and lymphocytes- including B cells, T cells, ILC3s and NK cells. Exhausted CD8^+^ T cells and macrophages are preferentially enriched in tumor. CD8^+^ T cells and macrophages show continuous activation pattern among distinct cell subsets.

The host immune system can be leveraged to treat tumor and improve outcomes for cancer sufferers [49]. Reprograming the tumor immune microenvironment (TME) to attract the right type of immune infiltrate e.g., by reversing exhaustion status, switching the macrophage transcriptome towards an M1 phenotype or deletion of pro-tumor macrophages is a promising anti-tumor therapeutic strategy. Evidently, this study provides a deeper understanding of the complex immune and endometrial epithelial cell types and their functional states within the endometrioid carcinoma ecosystem (Figure 9). It further provides reference points for future translational applications. Nevertheless, further studies are necessary to clarify the interplay between immune and endometrial epithelial cell types as well as their functional states in space and time.

## MATERIALS AND METHODS

### Ethics approval and consent to participate

This research project was approved by the Human Investigation Ethical Committee of Shanghai First Maternity and Infant Hospital. All patients participating in the study signed an informed consent.

### Histopathology and immunohistochemistry

Tissue samples from representative lesions were collected and fixed in 10% formalin. 5 μM slides were obtained from paraffin-embedded tissues and stained with hematoxylin and eosin for histopathological examination. For immunohistochemistry analysis, the “UltraVision Quanto Detection System HRP DAB” IHC kit (TL-125-QDH, Thermo Fisher Scientific) was used for the tyramide signal amplification according to the manufacturer’s protocol. Primary antibodies used in this assay are as follows: anti-Ckpan (GM351529, Gene Tech), anti-CD31 (M082329-2, Dako), anti-vimentin (VIM) (Y23037, Ventana), anti-CD3 (ab16669, Abcam), anti-CD20 (M0755, Dako), anti-CD68 (ab955, Abcam), anti-CD163 (ab156769, Abcam), and anti-CD8 (ab17147, Abcam), anti-Ki67 (M0350, Long Island Antibody), anti-p16 (F07961, Roche), anti-MSH6 (RMA-0770, MXB Biotechnologies). Images were taken and quantitative image analysis was performed using ImagePro software.

### Preparation of single-cell suspensions

Following resection, a representative tumor fragment and paratumor tissues (1cm from the boundary of tumor) were isolated and transferred rapidly to the laboratory for study (Supplementary Figure 1A). For patients EC4 and EC5, the tumor sizes were too big to acquire the paratumor tissues. Fresh tumor and paratumor tissue samples were initially cut into segments, then transferred to 10 ml digestion DMEM medium containing 0.2% collagenase type I/II (Thermo Fisher Scientific, USA) and DNAse I (Sigma, USA), and were incubated for 15 min at 37° C. The digested pieces were triturated with a 1 ml syringe plunger and passed through a 70μm cell strainer (Coring, USA). The resulting suspension was centrifuged at 300 g for 5 min, then resuspended in red blood cell lysis buffer (Solarbio, China) and incubated on ice for 5 min. After washing with 1x PBS, live cells were enriched using a Dead Cell Removal kit (Miltenyi Biotec, Germany) as per manufacturer’s instructions. Enriched live cells were washed and counted using a hemocytometer with trypan blue. Cells were then resuspended in PBS containing 0.04% BSA at a concentration of 1 × 10^6^ cells/ml with a viability of > 80% as determined with the Countess. Overall, the entire dissociation procedure took about 2 h from obtaining samples to generating single-cell suspensions. The single-cell suspensions were then run on the Chromium 10X device (10 × Genomics, USA).

### 10× library preparation and sequencing

Single-cell library preparation was carried out using Chromium Single cell 3’ Reagent v2 Kits (10 × Genomics, USA) according to the manufacturer’s protocol. Cells were loaded on the Chromium Single Cell Controller Instrument to generate single cell gel beads in emulsions (GEMs). Next, reverse transcription was performed, cDNA was cleaned up with DynaBeads Myone Silane Beads (Thermo Fisher Scientific, USA), and was then amplified by PCR with appropriate cycles. Subsequently, the amplified cDNA was fragmented, end-repaired, A-tailed, index adaptor-ligated and subjected to library amplification. Then these libraries were sequenced on the HiSeq X Ten instruments (Illumina, USA) and 150 bp paired-end reads were generated.

### Single-cell RNA-seq data preprocessing.

The Cell Ranger software pipeline (version 2.2.0, https://support.10xgenomics.com/single-cell-gene-expression/software/downloads/2.0) provided by 10xGenomics was used to process reads. Fastq files generated from Illumina sequencing output were mapped to the human reference genome (GRCh37) and transcriptome using the STAR aligner, and then read count matrices were generated by counting unique molecular identifiers (UMIs). Finally, we generate a gene-barcode matrix containing the barcoded cells and gene expression counts. We combine multiple libraries and generate normalized aggregate data across samples using the cellranger aggregation function.

We imported the count data into the Seurat (version 2.3.4) R package for quality control. We first excluded genes detected in < 3 cells and cells where < 100 genes had nonzero counts. We further discarded low-quality cells that had > 5% mitochondrial genes.

Library size normalization was performed in Seurat on the filtered matrix to obtain the normalized count. Additional cell–cell normalization was performed using the LogNormalize method, and inherent variation caused by mitochondrial gene expression and the number of unique molecular identifiers (UMIs) per cell was regressed out. Gene expression matrices were normalized to total cellular read count and to mitochondrial read count using linear regression as implemented in Seurat’s ScaleData function. Before incorporating a sample into our merged dataset, we individually inspected the cells-by-genes matrix of each as a Seurat object.

### Dimensionality reduction and clustering

Following normalization, highly variable genes we identified using the Seurat FindVariableGenes function. This function calculates the mean expression and dispersion for each gene, then places genes into 20 bins based on expression. Biologically variable genes were then captured as having a normalized log mean expression between 0.125 and 8, and a dispersion exceeding 1.

The generated variable genes were used to perform principle component analysis (PCA). We then used the first 20 principle components (PCs) to construct a two-dimensional representation of the data using t-distributed stochastic neighbor embedding (t-SNE) with perplexity 20. This representation was then used to visualize the data.

Clusters were identified from PCA-reduced expression data at a resolution of 1 using the Seurat "FindClusters" algorithm, which calculates the neighborhood overlap between every cell and its nearest neighbors. Graph-based clustering results were visualized in 2-dimension using t-SNE. Individual samples and sample groups were also visualized using t-SNE.

Cell clusters in the resulting two-dimensional representation were annotated to known biological cell types using canonical marker genes.

### Identification of cluster marker genes and differential expression analysis

The cluster-specific marker genes were identified using differential expression analysis. The difference between clusters was analyzed by “FindAllMarkers” function in the Seurat package. A marker gene was identified when it was expressed in a minimum of 25% of cells and at a minimum log fold change threshold of 0.25. In paired analyses, we identified differentially expressed genes (DEGs) if the absolute log_2_ expression fold change was ≥ 0.4 and the Benjamini–Hochberg adjusted *P* value was ≤ 0.01.

### Subclustering of the major cell types

To identify subclusters within epithelial, T, and myeloid cell types, we reanalyzed cells annotated to these three cell types separately. Briefly, first we get annotated clusters form cells form raw Seurat object using the “SubsetData” function of the Seurat package. We performed dimensionality reduction using PCA in each cell type on variable genes as described above. Using the graph-based clustering approach implemented in the “FindClusters” function of the Seurat package, with a conservative resolution of 1 and otherwise default parameters, each cell type was reclustered by its principle components. For visualization purposes, these informative principle components were converted into t-SNE plots as above. Gene expression data for subclusters are available in Additional File 3.

To identify marker genes for each of these subclusters within the immune cell types, we contrasted cells from that subcluster to all other cells of other subclusters using the Seurat FindMarkers function. Marker genes were required by log_2_FoldChange ≥ 1, adjust *P* value ≤ 0.01, and ranking top 100. When analyzing marker genes for several subclusters in aggregate, such as for tumor macrophages (myeloid clusters 0, 2, 4, 5, 6, 7 and 8 in Tumor), we simply combined the marker genes for all associated subclusters [12] (Additional File 4).

### Gene set enrichment analysis

Gene set enrichment analysis for differentially expressed genes was performed using Gene Ontology (biological process, cell component, and molecular function), and Kyoto Encyclopedia of Genes and Genomes (KEGG) pathway database. In a pairwise comparison between paratumor vs tumor data in Figure 6B, DEGs were identified by |log_2_FoldChange| ≥ 0.4 and adjust *P* value ≤ 0.01 (Additional File 1). In the pairwise comparison between paratumor vs tumor data in Figure 7E, 7F, DEGs were identified by |log_2_FoldChange| ≥ 0.25 and adjust *P* value ≤ 0.01 (Additional File 2). When performing pathway enrichment analysis in endometrial epithelial clusters in Figure 8E, 8F, up-expressed genes in per cluster were used with log_2_FoldChange ≥ 0.25 and adjust *P* value ≤ 0.01. We used the “clusterProfiler” function implemented in R packages to identify significant altered pathways. The z scores were computed from normalized -log_10_(*P* value) generated from the Fisher exact test. Pathway enrichment heatmap results were visualized using R package heatmap (version 1.0.12).

### Pseudo-time analysis

The monocle R package (version 2.10.1) was used to perform the trajectory analysis on the epithelial cells. Genes expressed in fewer than 3 cells were excluded, library size normalization was performed by the "estimateSizeFactors" function, and negative binomial over-dispersion was estimated for each gene using the "estimateDispersions" function. We selected genes that have mean expression > 0.5 and variance greater than the empirical dispersion (the best fit mean-dispersion trend-line). We used the DDRTree to do dimension reduction on the selected genes and then constructed a trajectory using the "orderCells" function.

### State analysis in T cells and macrophages

T cell exhaustion, T cell activation, anti-inflammatory (immunosuppressive) and pro-inflammatory (immunostimulatory) gene signatures were taken from Azizi et al. [26] and used for CD8^+^ T cells state analysis. M1, M2, monocyte activation and macrophage activation gene signatures were taken from Azizi et al. [26] and used for macrophage state analysis. In all cases, the intensity of expression of the signature in question was computed as the mean expression of the genes included in the signature.

### CNV estimation

The normalized scRNA-seq gene expression matrices were used to estimate CNV profiles with inferCNV R package as previously described [50]. Initial CNVs were estimated by sorting genes based on their chromosomal location and applying a moving average of gene expression with a window size of 101 genes. The expression was then centered to zero by subtracting the mean. The stromal cells were assigned as "normal" cells and background for analysis. The de-noising was carried out to generate the final CNV profiles. The CNV score of each cell was calculated as quadratic sum of CNVregion.

### TCGA data analysis

Pre-processed gene expression data (fragments per kilobase per million fragments) RNaseq v3 mRNA expression data as well as clinical parameters for tumors and normal solid tissue, for endometrial cancer (TCGA-UCEC), using the Bioconductor TCGAbiolinks package (version 2.10.5). In order to assess the expression and prognostic value of a gene set, the average log-normalized expression of selected genes was computed. The samples were further stratified as “low expression” group and “high expression” group by the average expression of immune cell marker genes. The statistical analysis was performed by the R package ‘survival’ (version 3.1.8), and survival curves were fitted by the survfit function and the difference between high and low expression group was test by survdiff.

### Statistical analysis

Data were analyzed with GraphPad Prism software. For comparison between two groups, statistical evaluation was done by two-tailed Student’s t-test. For all statistical tests, the *P* values <0.05 were considered statistically significant. All error bars show standard error of the mean (SEM).

### Data availability

The sequencing raw data have been deposited on SRA database. The SRA accession number is PRJNA650549, and the SRA records link is: https://www.ncbi.nlm.nih.gov/sra/PRJNA650549

## Supplementary Material

Supplementary Figures

Additional File 1

Additional File 2

Additional File 3

Additional File 4
