# Supplementary material for: Phenotyping of immune and endometrial epithelial cells in endometrial carcinomas revealed by single-cell RNA sequencing
Source: Aging (Albany NY). 2021 Jan 10;13(5):6565–91. doi: 10.18632/aging.202288 (PMC7993685; doi:10.18632/aging.202288)
Supplement: Supplementary Figures [file aging-13-202288-s001.pdf]

SUPPLEMENTARY FIGURES

A

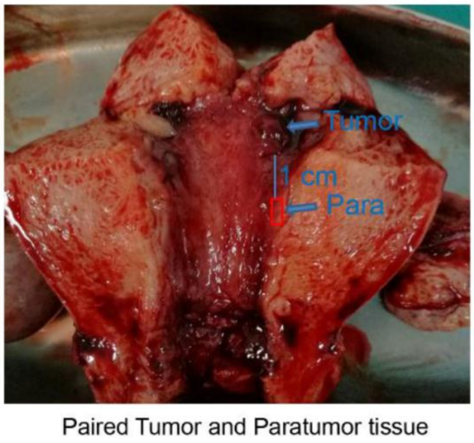

B

	EC1	EC2	EC3	EC4	EC5
Paratumor	+	+	+	-	-
Tumor	+	+	+	+	+
Age (year)	51	68	42	55	56
Histological type	endometrioid	endometrioid	endometrioid	endometrioid	endometrioid
Histological grade	G1	G1	G1	G1	G1
FIGO stage	I	I	I	I	II
Lymph node metastasis	negative	negative	negative	negative	negative
Myometrial invasion	<50%	<50%	<50%	<50%	>50%
ER	60%+	60%+	90%+	80%+	70%+
PR	60%+	70%+	90%+	70%+	20%+
PTEN	-	-	-	-	-
p53	WT	WT	WT	WT	WT
p16	patchy+	patchy+	-	patchy+	patchy+
Ki67	10%+	10%+	5%+	30%+	25%+
MLH1	+	+	+	+	+
MSH2	+	+	+	+	+
MSH6	+	+	+	+	-
PMS-2	+	+	+	+	+
POLE	WT	WT	WT	WT	WT

C

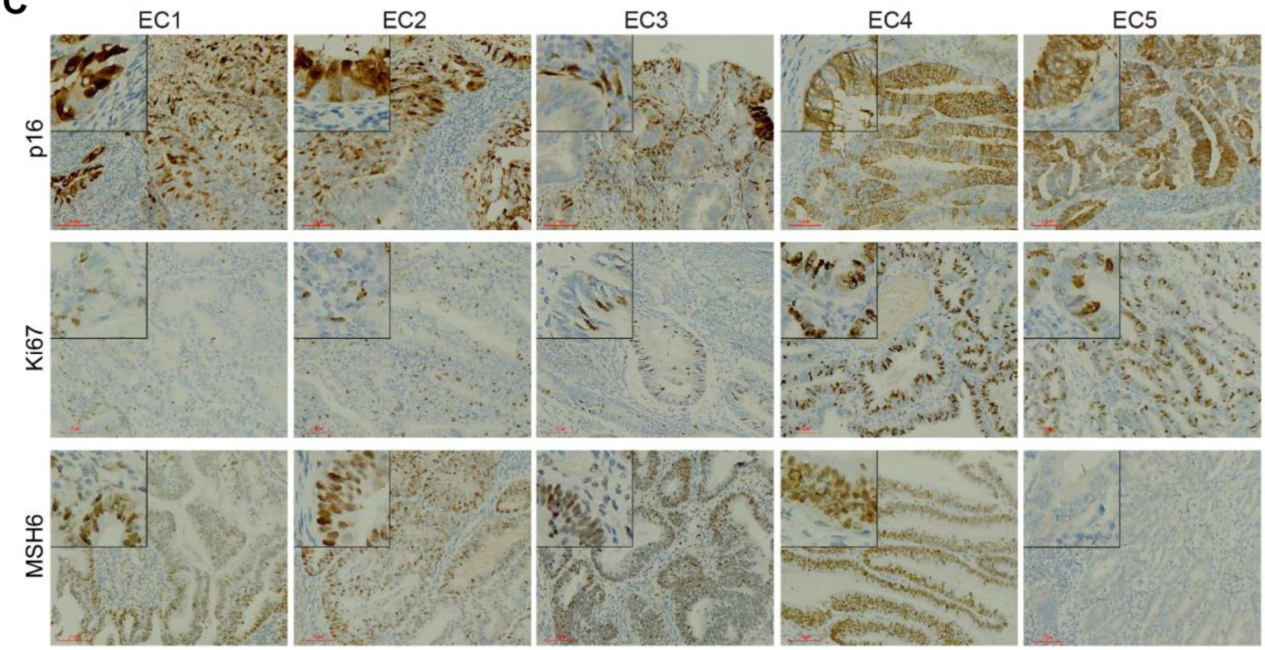

Supplementary Figure 1. Additional details of the samples, related to Figure 1. (**A**) Representative image of paired tumor and paratumor tissues (1cm from the boundary of tumor) acquisition. (**B**) Samples obtained from 5 EC patients, clinicopathological characteristics of the 5 patients, expression of phenotype-related genes (ER, PR, PTEN, p53, p16, Ki67, MLH1, MSH2, MSH6, and PMS2) detected by immunohistochemical analysis and genotyping of POLE gene. ER and PR values denote proportion of tumor cells that express the receptor. (-) Means negative expression, while (+) means positive expression. WT in p53 means positive normal phenotype expression. P16 expression was estimated in the epithelial cells. (**C**) IHC staining images of p16, Ki67 and MSH6 in tumor slides isolated from endometrial carcinoma sections. Scale bars, 80 µm.

A

	EC1-P	EC1-T	EC2-P	EC2-T	EC3-P	EC3-T	EC4	EC5
Estimated Number of Cells	3,880	4,004	1,951	2,352	5,679	2,011	7,077	4,564
Mean Reads per Cell	159,424	139,275	294,698	234,303	139,015	379,740	106,813	141,356
Median Genes per Cell	1,930	2,518	2,586	1,688	2,917	4,194	2,695	2,043
Number of Reads	618,566,534	557,658,646	574,957,144	551,081,759	789,467,095	763,659,050	755,917,824	645,152,798
Valid Barcodes	98.00%	97.80%	97.90%	97.90%	97.70%	97.90%	97.20%	97.60%
Sequencing Saturation	87.10%	80.10%	86.90%	91.10%	77.60%	78.50%	71.50%	83.40%
Q30 Bases in Barcode	95.90%	95.80%	96.70%	96.70%	96.60%	96.60%	96.50%	96.20%
Q30 Bases in RNA Read	91.30%	90.90%	93.60%	93.00%	93.10%	93.30%	92.80%	93.00%
Q30 Bases in UMI	95.60%	95.50%	96.60%	96.60%	96.60%	96.60%	95.90%	95.40%
Reads Mapped to Genome	95.00%	94.50%	96.40%	95.90%	95.60%	95.70%	95.50%	95.20%
Reads Mapped Confidently to Genome	92.60%	92.10%	94.30%	93.70%	93.00%	92.90%	92.90%	91.70%
Reads Mapped Confidently to Intergenic Regions	3.90%	3.80%	3.40%	3.40%	4.10%	4.30%	3.90%	5.20%
Reads Mapped Confidently to Intronic Regions	19.30%	21.80%	20.30%	21.80%	30.80%	24.90%	19.40%	16.80%
Reads Mapped Confidently to Exonic Regions	69.40%	66.50%	70.60%	68.50%	58.10%	63.70%	69.60%	69.70%
Reads Mapped Confidently to Transcriptome	66.20%	63.30%	66.00%	65.20%	54.60%	60.20%	65.90%	66.30%
Reads Mapped Antisense to Gene	0.90%	0.90%	2.00%	1.20%	1.30%	1.10%	1.00%	0.80%
Fraction Reads in Cells	73.90%	75.90%	57.50%	65.10%	73.00%	63.00%	70.60%	73.40%
Total Genes Detected	22,201	22,409	21,041	21,492	24,591	24,114	23,044	23,081
Median UMI Counts per Cell	5,795	8,712	8,183	4,920	9,247	17,964	10,962	6,377

B

	EC1-P	EC1-T	EC2-P	EC2-T	EC3-P	EC3-T	EC4-T	EC5-T
Epithelial cells	1,446	1,692	1,661	558	423	547	6,267	958
Endothelial cells	0	0	0	70	486	91	0	108
Fibroblasts	93	27	23	46	2,618	395	58	158
T-cells	1,953	645	70	811	1,545	255	235	2,202
B-cells	65	0	0	0	0	0	31	215
Myeloid cells	232	1,524	149	833	511	467	435	877
Total	3,789	3,888	1,903	2,318	5,583	1,755	7,026	4,518

Supplementary Figure 2. Quality control and cell type numbers of each sample, related to Figure 1. (**A**) Aggregated quality control and metric information for each sequencing sample. (**B**) Cell number of major cell types in each sample.

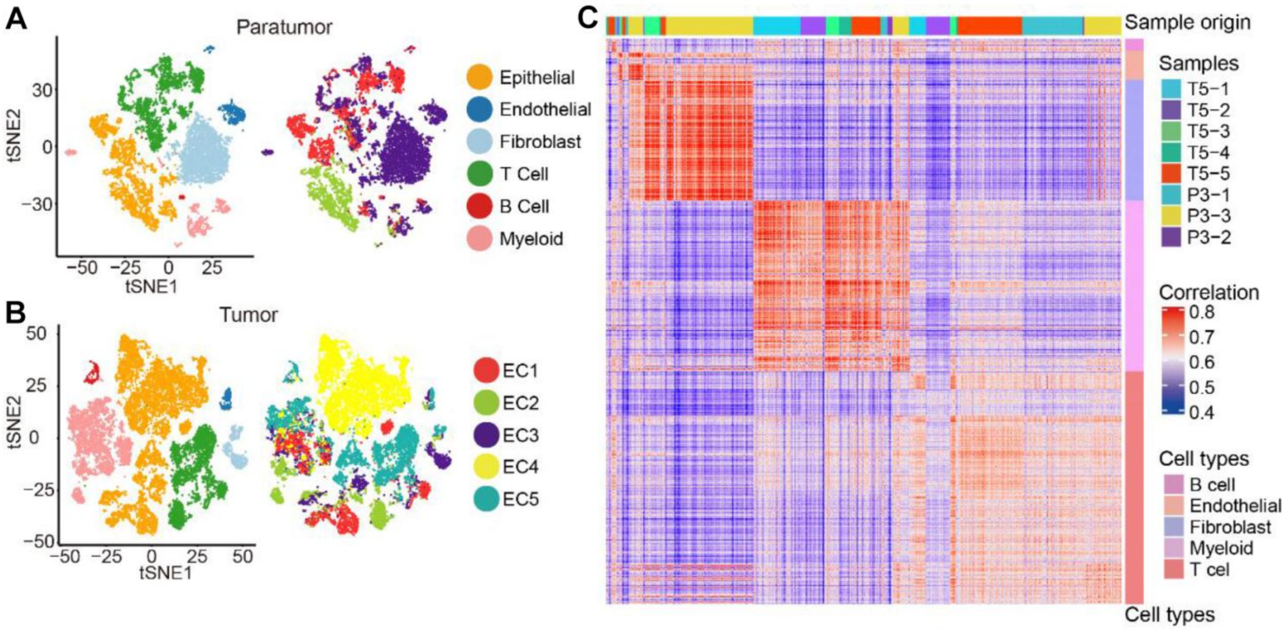

Supplementary Figure 3. Integrated clustering of paratumor and endometrial tumor samples and centred correlation matrix for stromal single cells, related to Figure 2. (**A**, **B**) t-SNE projection of the 11,275 cells from 3 integrated paratumor samples (**A**) and 19,505 cells from 5 integrated tumor samples (**B**). Each cell type (left) and the corresponding patient (right) is shown by different color. (**C**) Centred correlation (Pearson's r) matrix for stromal single cells from different samples. Each row and column represents single cells.

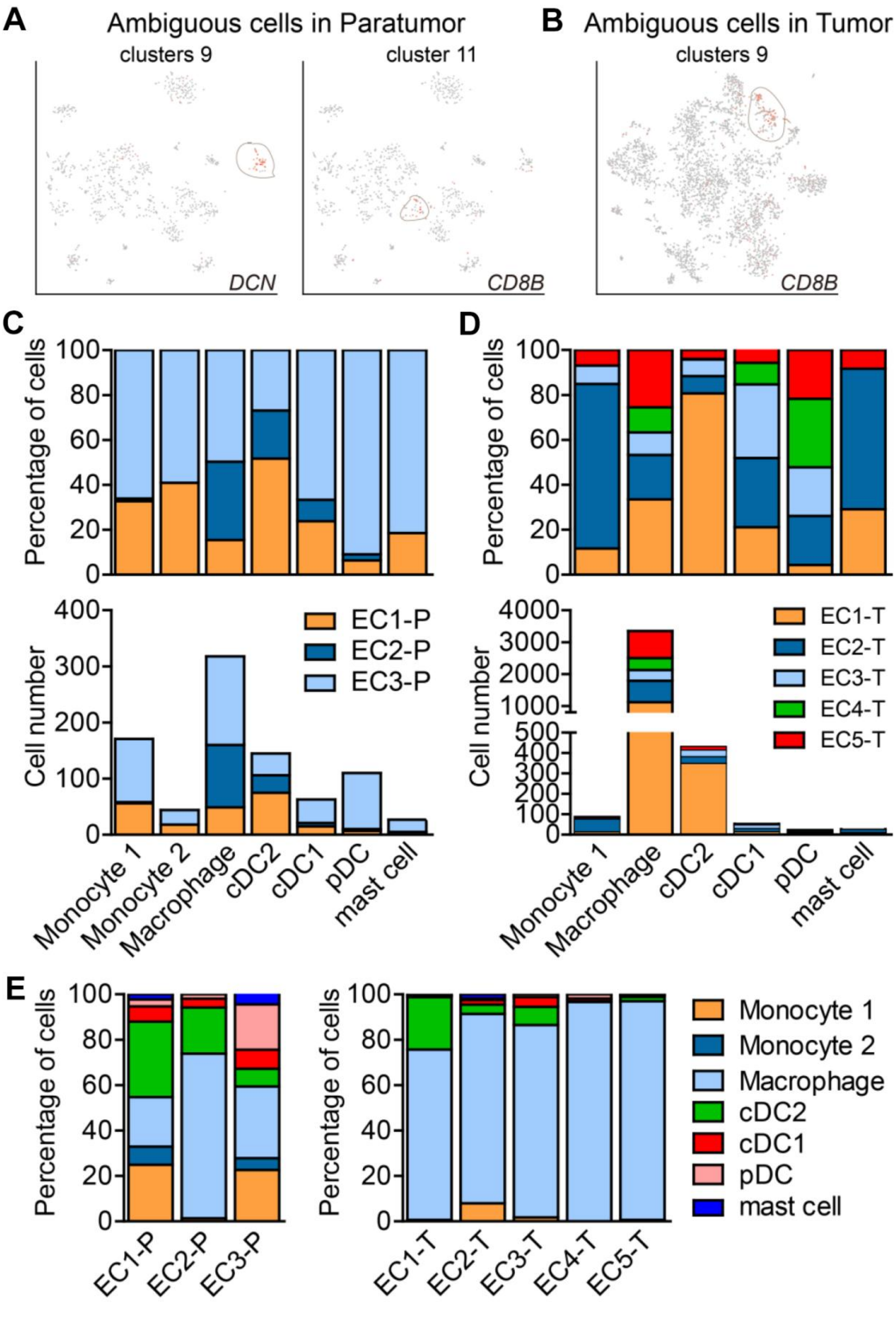

Supplementary Figure 4. Ambiguous cells in myeloid cell subclustering and myeloid cell subtypes distribution, related to Figure 3. (**A**, **B**) t-SNE plot, color-coded for relative expression of marker genes for non-myeloid cells (ambiguous cells) in Paratumor (**A**) and Tumor (**B**). (**C**, **D**) For each cell subtype: the cell fractions and numbers originating from each of the 3 paratumor (**C**) and 5 tumor (**D**) samples are shown. (**E**) The fractions of the myeloid cell subtypes in each sample.

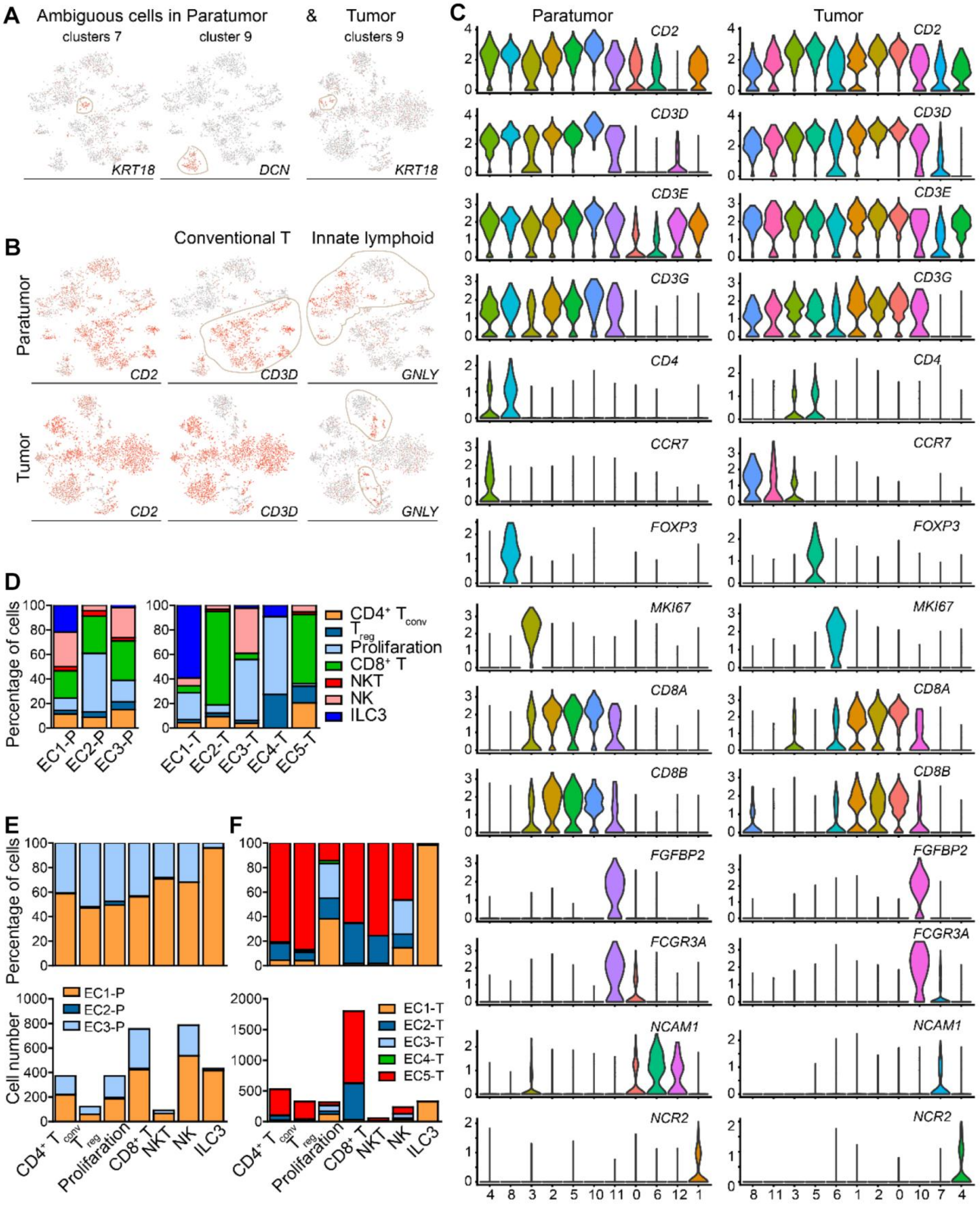

Supplementary Figure 5. Subclustering of paratumor and tumor lymphocytes, related to Figure 5. (**A**) t-SNE plot, color-coded for relative expression of marker genes for non-lymphocytes (ambiguous cells) in Paratumor and Tumor. (**B**) t-SNE plot, color-coded to show the relative expression (gray to red) of marker genes for the conventional T cells and innate lymphoid cells. (**C**) Violin plots displaying the expression profile of known marker genes of lymphoid subtypes in the cell clusters in Paratumor (left panel) and Tumor (right panel). The y axis shows the normalized expression. (**D**) The fractions of the 7 lymphoid subtypes in each sample. (**E**, **F**) For each cell subtype: the cell fractions and numbers originating from each of the 3 paratumor (**E**) and 5 tumor (**F**) samples are shown.
